# Temperature Affects the Use of Storage Fatty Acids as Energy Source in a Benthic Copepod (*Platychelipus littoralis*, Harpacticoida)

**DOI:** 10.1371/journal.pone.0151779

**Published:** 2016-03-17

**Authors:** Eva Werbrouck, Dirk Van Gansbeke, Ann Vanreusel, Marleen De Troch

**Affiliations:** Department of Biology, Marine Biology Research Group, Ghent University, Ghent, Belgium; Stazione Zoologica Anton Dohrn, Naples, ITALY

## Abstract

The utilization of storage lipids and their associated fatty acids (FA) is an important means for organisms to cope with periods of food shortage, however, little is known about the dynamics and FA mobilization in benthic copepods (order Harpacticoida). Furthermore, lipid depletion and FA mobilization may depend on the ambient temperature. Therefore, we subjected the temperate copepod *Platychelipus littoralis* to several intervals (3, 6 and 14 days) of food deprivation, under two temperatures in the range of the normal habitat temperature (4, 15°C) and under an elevated temperature (24°C), and studied the changes in FA composition of storage and membrane lipids. Although bulk depletion of storage FA occurred after a few days of food deprivation under 4°C and 15°C, copepod survival remained high during the experiment, suggesting the catabolization of other energy sources. Ambient temperature affected both the degree of FA depletion and the FA mobilization. In particular, storage FA were more exhausted and FA mobilization was more selective under 15°C compared with 4°C. In contrast, depletion of storage FA was limited under an elevated temperature, potentially due to a switch to partial anaerobiosis. Food deprivation induced selective DHA retention in the copepod’s membrane, under all temperatures. However, prolonged exposure to heat and nutritional stress eventually depleted DHA in the membranes, and potentially induced high copepod mortality. Storage lipids clearly played an important role in the short-term response of the copepod *P*. *littoralis* to food deprivation. However, under elevated temperature, the use of storage FA as an energy source is compromised.

## Introduction

Many aquatic habitats are shaped by a pulsed seasonal primary production which implies a restricted food availability for various herbivores at certain times. One strategy to cope with these natural cycles of food intake is the efficient storage and utilization of energy reserves [1,2]. Lipids contain the highest energy content compared with carbohydrates and proteins [3] and are a major energy storage product of cladocerans and copepods [4]. Storage lipids are especially well studied in copepods inhabiting extreme environments i.e. polar and deep-living copepods. In these species they provide crucial energy for reproduction, periods of low food supply, obtaining food, escaping predation and for vertical migration [3,5]. In situations of negative energy balance such as fasting, storage lipids are metabolized so that fatty acids (FA) become available as metabolic fuel to prolong survival [4,6]. Whether the process of FA mobilization from storage lipids is selective, remains open to debate [7]. Furthermore, in case preferential FA mobilization is reported, the pattern differs among crustaceans with respect to the preferential utilization [8] or retention [9] of polyunsaturated FA (PUFA). Especially, the dynamics of the essential FA, EPA (20:5ω3) and DHA (22:6ω3) in food deprived crustaceans, have received considerable attention [10–12] in view of their beneficial physiological effects in multiple consumers [13,14].

In contrast to planktonic crustaceans such as cladocerans [10,15] and freshwater copepods [8], almost nothing is known about the dynamics of storage lipids and, in particular, the FA mobilization in harpacticoid copepods. The limited knowledge on storage lipids in harpacticoid copepods, but see [16,17], contrasts with their dominant role in benthic food webs. After free-living nematodes, harpacticoid copepods are usually the second most abundant metazoan taxon of the meiobenthos and their importance as prey for higher trophic levels has been demonstrated [18]. Furthermore, the trophic position of copepods as first-level consumer [19] and their potential FA upgrading capacity [20], suggest their importance in the transfer of energy and essential FA to higher trophic levels.

Therefore we subjected a temperate harpacticoid copepod to several intervals of food deprivation (3, 6 and 14 days) and screened the membrane and storage lipids for their associated FA content and composition. Starvation studies may indicate which energy sources are utilized by crustaceans under specific conditions and they provide clues on the biochemical pathways involved in these processes [1]. Our species of interest is the sluggish, non-swimming harpacticoid copepod *Platychelipus littoralis* (Family Laophontidae Brady 1880). Due to its restricted mobility, this species is directly linked to the local conditions on a microspatial scale [21]. Environmental temperature strongly affects the metabolic rate in ectotherms [22] and can affect the rate of lipid breakdown [23], and may determine which FA are preferentially metabolized during fasting [8]. Therefore, the starvation response of *P*. *littoralis* was studied under two temperatures, in the range of the normal habitat temperature (4, 15°C), and also under an elevated temperature (24°C). This strategy allowed to investigate:

1. the importance of storage lipids for copepod survival in response to food deprivation,
2. whether selective FA mobilization from storage lipids occurs in *P*. *littoralis* specimens and if it is temperature-dependent,
3. the changes in membrane FA content and composition, which inform on the copepod’s thermal adaptation and physiological condition.

The starvation response of *P*. *littoralis* will be interpreted in a broader ecological context with respect to its natural habitat.

## Material and Methods

### Experimental set-up

The harpacticoid copepod *Platychelipus littoralis* was collected at the Westerschelde estuary (51°21’N, 3°43’E, the Netherlands) from the top sediment layer in a small intertidal creek at the Paulina saltmarsh (May 2015 –average temperature: 12±1°C). Permits for the field work were approved and obtained by Provincie Zeeland, the Netherlands; Directie Ruimte, Milieu en Water. Within hours after collection, copepods were extracted alive using sediment decantation. Subsequently, adult specimens were randomly collected, excluding ovigerous females, with a glass pasteur pipette using a Wild M5 binocular. All copepods were kept overnight in glass jars with some sediment aliquots at 15±1°C prior to the start of the experiment. Triplicates of 20 and 100 copepods were stored at -80°C for further dry weight and lipid fractionation (FA analysis), respectively. After thawing, samples for dry weight measurements were obtained by rinsing copepods several times in MilliQ water to remove adhering particles and by transferring them to pre-weighted aluminum cups (6 x 2.5 mm, 20 ind.). Samples were kept overnight in an oven at 100°C and were weighted using a microbalance (Mettler, Toledo, XP56). Units of 120 copepods were stored in Petri dishes (surface area = 26.4 cm^2^, 20 ml) and incubated at 4°C, 15°C and 24°C (±1°C), under a 12:12 h light-dark regime (25 to 50 μmol photons m^-2^ s^-1^). The copepods were kept in artificial seawater (Instant Ocean synthetic salt, salinity: 25, filtered over 0.2 μm Millipore filters) in order to guarantee complete food deprivation. After one day of starvation, copepods were transferred to new Petri dishes and (acclimated) artificial seawater to remove their fecal pellets. This procedure was performed every three days over the course of the experiment to compensate for potential evaporation and salinity changes, especially under 24°C. After 3, 6 and 14 days of starvation three units were randomly chosen at each incubation temperature. Copepod mortality was recorded and surviving copepods were stored at -80°C for later dry weight measurements and lipid fractionation (FA analysis). The experiment was terminated when survival rate was around 50% in one of the three temperature treatments.

### Lipid fractionation and FA analysis

Total lipids of copepods were extracted with a modified Bligh and Dyer extraction [24]. Subsequently, the total lipid extract was fractionated on a silicic acid column (Merck) into different polarity classes by sequential eluting with chloroform (containing neutral lipids, NLFA), acetone and methanol (containing polar lipids, PLFA) [25]. Derivatization of PLFA in the methanol fraction to FAMEs (fatty acid methyl esters) was obtained using a mild alkaline methanolysis as in [26]. FA associated with the acetone and chloroform fractions were derivatized using a modified method after [27]. Here, the boron trifluoride-methanol reagent was replaced by a 2.5% H_2_SO_4_-methanol solution, since the BF_3_-methanol can cause artifacts or loss of PUFA [28]. FAME of 19:0 (Fluka 74208) was added as internal standard. FAMEs were concentrated to 200 μl hexane and thereafter, injected and analyzed using a Hewlet Packard 6890N gas chromatograph coupled to a HP 5973 mass spectrometer as in [20]. The samples were run in splitless mode injecting 1μl at an injector temperature of 250°C using an HP88 column (Agilent J&W; Agilent). The oven temperature was programmed at 50°C for 2 min, followed by a ramp at 25°C min^-1^ to 175°C and then a final ramp at 2°C min^-1^ to 230°C with a 4 min hold. The FAMEs were identified by comparison with the retention times and mass spectra of authentic standards and mass spectral libraries (WILEY, own library) and analyzed using the software MSD ChemStation (Agilent Technologies). Quantification of individual FAMEs was accomplished by the use of a component FAME and BAME (Bacterial Acid Methyl Esters) mix (Supelco #47885 and #47080 respectively, Sigma-Aldrich) and completed with additional standards (Larodan). The quantification function of each individual FAME was obtained by linear regression of the chromatographic peak areas and corresponding known concentrations of the standards (ranging from 25 to 200 μg ml^-1^). Shorthand FA notations of the form A:BωX were used, where A represents the number of carbon atoms, B gives the number of double bonds, and X gives the position of the double bond closest to the terminal methyl group. FA concentrations were standardized to mg dry weight (mg DW).

### Data analysis

Copepod survival (%), membrane FA content (summed FA mass fraction from polar lipids) and storage FA content (summed FA mass fraction from neutral lipids) were log transformed in order to fulfill the assumptions for normality and homogeneity of variance which are required for the ANOVA tests (IBM SPSS Statistics Version 22). In case of significant differences, Tukey HSD *post*-*hoc* tests were applied to detect pairwise differences, using 95% confidence limits. In case the assumptions were not met after log transformation, non-parametric tests (PERMANOVA) were performed using Primer 6 Version 6.1.11 and 1.0.1 [29].

Prior to the statistical analysis, relative FA concentrations (%) of membrane and storage lipids were arcsine square root transformed in order to meet the assumptions for normality and homogeneity of variance which are required for the principal component analysis (PCA) (Primer 6 Version 6.1.11 and 1.0.1). Only FA with less than 20% of zero values were included in the PCA analysis. PCA for membrane and storage FA composition were run separately but included all time measurements (day 0, starvation after 3, 6 and 14 days). Eigenvalue variation explained by PC1 was 57% and 69% for the membrane and storage FA composition, respectively. In line with previous work [8,30], the sample scores on the PC1 were further used for statistical analysis as the new variable ‘PC1score’, as they represent the major trends in FA composition. Subsequently, the PC1scores for membrane and storage FA composition were related with each individual FA (proportion) by calculating the Spearman’s rho correlation coefficient (IBM SPSS Statistics Version 22).

One-way tests compared membrane and storage FA contents, PC1score values of membrane and storage FA composition in copepods prior to (day 0) and after short-term food deprivation (3 days) under different temperatures. Two-way tests for the factors temperature (4, 15 and 24°C) and time (3, 6 and 14 days) were conducted for copepod survival, membrane and storage FA contents, PC1score values of membrane and storage FA composition, to reveal long-term food deprivation effects. Ultimately, dynamics in EPA and DHA content were tested with two-way tests. In particular, two-way tests compared the EPA and DHA content associated with membrane or storage lipids in copepods prior to (day 0) and after short-term food deprivation (3 days) under different temperatures. Furthermore, any long-term effects of food deprivation on the EPA and DHA content associated with membrane or storage lipids were revealed with two-way tests for the factors temperature and time.

## Results

### Copepod survival

Dead copepods were already observed after 3 days of food deprivation under 24°C (survival 97±4%). However, copepod survival decreased only significantly after 14 days of starvation at 24°C (56±9%) compared with 4°C (99±2%) and 15°C (95±3%) (all p <0.01 for temperature; time and their interaction).

### Membrane and storage FA content

**Fig 1:**
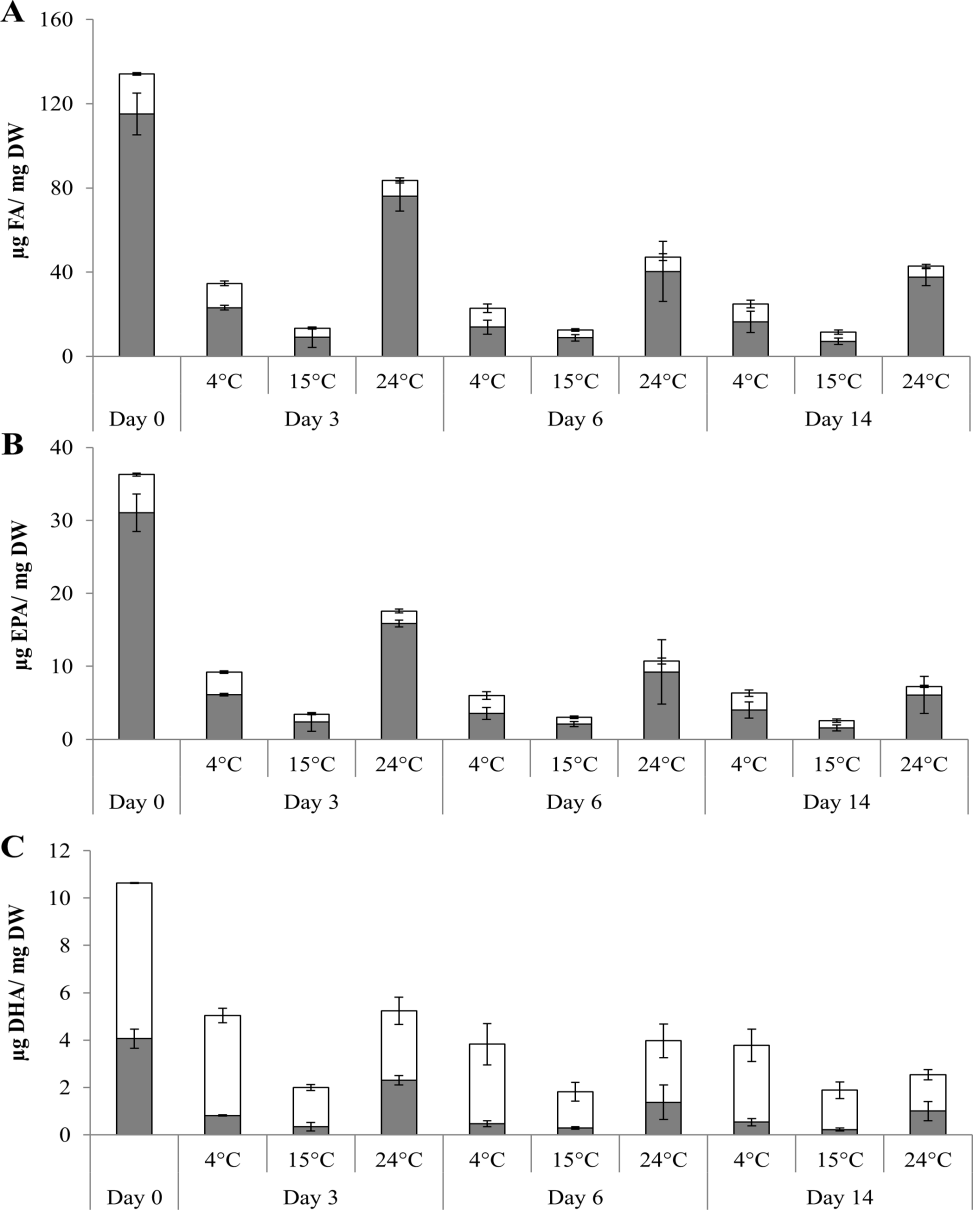
The lipid profile of the copepod *P*. *littoralis* prior (day 0) and after 3, 6 and 14 days of food deprivation at different temperatures (4, 15 and 24°C). (A) the storage and membrane FA content (μg FA/ mg DW) (±SD), (B) EPA content (20:5ω3; μg EPA/ mg DW) (±SD), (C) DHA content (22:6ω3; μg DHA/ mg DW) (±SD) both associated with the storage and membrane lipids. Grey and white bars represent storage and membrane lipids, respectively.

At the onset of the experiment, copepods contained a membrane FA content of 18.9±0.6 μg FA/ mg DW which decreased significantly after short-term (3 days) food deprivation under each temperature (p <0.01) (Fig 1A). Moreover, the membrane FA content differed significantly among all temperature treatments (pairwise tests, all p <0.05) with the highest content at 4°C (11.5±1.1 μg FA/ mg DW), followed by 24°C (7.4±1.2 μg FA/mg DW) and 15°C (4.3±0.4 μg FA/ mg DW). Prolonged starvation (between day 3 and day 14) further reduced the membrane FA content for all temperatures, while the significant effect of ambient temperature remained (p <0.01 for temperature; p <0.05 for time; no interaction).

The original storage FA content (115.1±9.9 μg FA/ mg DW) decreased significantly after 3 days of starvation (p <0.01) with the strongest reduction observed at 15°C (9.1±4.8 μg FA/ mg DW), followed by 4°C (23.1±1.1 μg FA/ mg DW) and 24°C (76.1±7.1 μg FA/ mg DW) (pairwise tests, all p <0.01) (Fig 1A). Prolonged starvation (from day 3 to day 6) further reduced the storage FA content under 4°C and 24°C but the overall temperature effect remained (all p <0.01 for temperature; time and their interaction).

### Membrane and storage associated EPA and DHA content

Short-term food deprivation reduced the original membrane EPA and DHA content (5.3±0.2 μg EPA/ mg DW and 6.6±0.02 μg DHA/ mg DW) significantly under each temperature (pairwise tests, all p <0.05) (Fig 1B and 1C). The smallest decrease was observed at 4°C (3.1±0.2 μg EPA/ mg DW and 4.2±0.3 μg DHA/ mg DW) followed by 24°C (1.7±0.3 μg EPA/ mg DW and 2.9±0.6 μg DHA/ mg DW) and 15°C (1.1±0.06 μg EPA/ mg DW and 1.7±0.1 μg DHA/ mg DW) (pairwise test, all p <0.05) (Fig 1B and 1C). Prolonged starvation further reduced the membrane EPA and DHA content, but the significant temperature effect remained (both p <0.01 for temperature; both p <0.05 for time; no interaction).

The original storage EPA and DHA content (31.0±2.6 μg EPA/ mg DW and 4.1±0.4 μg DHA/ mg DW) were significantly reduced after short-term food deprivation under each temperature (pairwise tests, all p <0.05) (Fig 1B and 1C). The strongest EPA and DHA reductions were observed at 15°C (2.4±1.3 μg EPA/ mg DW and 0.3±0.2 μg DHA/ mg DW) followed by 4°C (6.1±0.2 μg EPA/ mg DW and 0.8±0.02 μg DHA/ mg DW) and 24°C (15.9±0.5 μg EPA/ mg DW and 2.3±0.2 μg DHA/ mg DW) (pairwise tests, all p <0.01). However, long-term starvation equalized the storage EPA and DHA content between 4°C (4.0±1.1 μg EPA/ mg DW and 0.5±0.2 μg DHA/ mg DW) and 24°C (6.1±2.5 μg EPA/ mg DW and 1.0±0.4 μg DHA/ mg DW) compared with 15°C (1.6±0.4 μg EPA/ mg DW and 0.2±0.06 μg DHA/ mg DW) (all p <0.01 for temperature and time; both p <0.05 for interaction).

### Membrane FA composition

**Fig 2:**
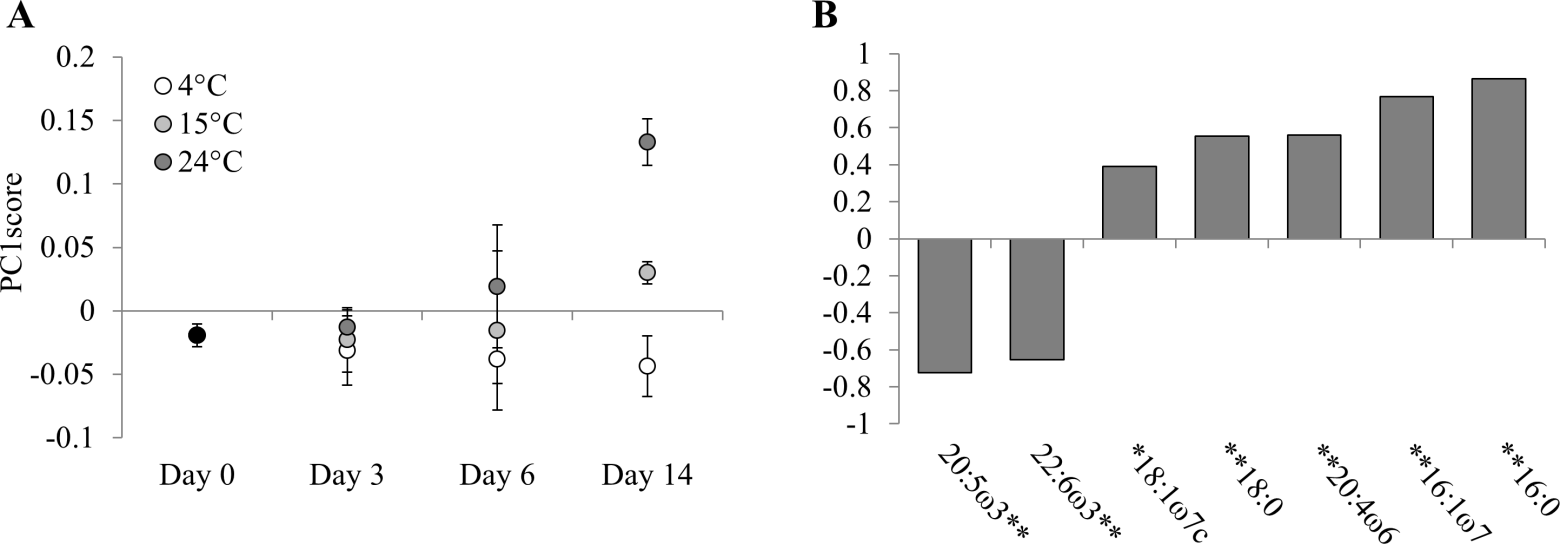
Membrane FA composition of the copepod *P*. *littoralis* prior (day 0) and after 3, 6 and 14 days of food deprivation at different temperatures (4, 15 and 24°C). (A) The PC1score values (±SD) for membrane lipids are displayed with (B) the Spearman’s rho correlation coefficients of individual FAs (%) used for the PCA with the correlation significance levels (*p <0.05; **p <0.01).

First, the original membrane FA composition remained unchanged after 3 days of food deprivation as indicated by the membrane PC1score values (Fig 2A). Thereafter, alterations appeared but they were temperature-dependent (p <0.01 for temperature and time; p <0.05 for interaction). In particular, long-term exposure under 15°C and 24°C, significantly altered the original membrane FA composition, while no significant changes were observed under 4°C, likely due to the large standard error values (pairwise tests, both p <0.05).

Prior to the experiment, the membrane FA composition was dominated by HUFA (highly unsaturated FAs i.e. FA ≥ 20 carbon atoms and ≥ 3 double bonds; 67.4±0.7%), followed by SFA (saturated FAs; 22.0±0.5%) and MUFA (mono unsaturated FAs; 10.3±0.3%) (Table 1). Food deprivation under cold conditions maintained the HUFA%, while a subtle increase in MUFA% at the expense of SFA% occurred. These shifts in FA classes were caused by the increase in relative concentrations of 18:1ω7c and DHA at the expense of 16:0 and 18:0 (Table 1), and the significant contributions of DHA, 16:0 and 18:0 (all p <0.01) also appeared from Fig 2B. In contrast, exposure to 15°C and 24°C increased the SFA% and MUFA% at the expense of HUFA% (Table 1). Under 15°C, the shifts in FA classes were caused by the increase in relative concentrations of 16:0 (after an initial decrease), 18:0, 18:1ω7c, 20:4ω6 and DHA at the expense of EPA (Table 1). This was largely confirmed by the significant contributions of 16:0, 18:0, 20:4ω6, EPA (all p <0.01) and 18:1ω7c (p <0.05) on Fig 2B. Under 24°C, the changes in membrane FA composition were most pronounced as HUFA% dropped to 54.8±1.6% by the end of incubation. Initially, increases in relative concentrations of 18:0, 18:1ω7c, 20:4ω6 and DHA, at the expense of EPA were observed (Table 1). However, towards the end of the experiment, the relative concentration of 16:0 and 16:1ω7 increased while DHA decreased substantially (all p <0.01) (Fig 2B).

**Table 1:** Membrane FA composition (% ±SD) of the copepod *P*. *littoralis* prior (day 0) and after 3, 6 and 14 days of food deprivation at different temperatures (4, 15 and 24°C); tr, traces (<1%); other FAs, sum of all FAs <1% in all treatments.

		4°C			15°C			24°C		
(%)	Day 0	Day 3	Day 6	Day 14	Day 3	Day 6	Day 14	Day 3	Day 6	Day 14
14:0	tr	tr	_	tr	_	_	_	_	_	1.1±1.0
16:0	16.5±0.3	15.0±1.2	14.7±0.3	13.6±1.5	14.5±1.3	15.4±3.1	17.4±0.5	16.1±1.0	16.8±2.9	19.3±0.4
16:1ω7	5.0±0.3	5.0±1.0	4.6±0.5	4.9±0.5	4.8±0.8	4.8±1.4	5.9±0.7	4.6±0.2	4.9±0.6	8.8±1.8
18:0	4.9±0.2	4.3±0.3	4.0±0.2	3.7±0.1	5.9±0.3	6.2±0.3	6.1±0.4	6.8±0.1	7.5±0.4	6.7±0.2
18:1ω7c	4.6±0.2	6.1±0.4	6.7±0.8	7.8±0.2	6.2±0.6	6.9±0.8	7.5±0.4	5.5±0.2	6.4±0.2	9.4±2.1
20:4ω6	1.6±0.1	1.6±0.1	1.7±0.1	1.7±0.05	2.2±0.3	2.3±0.1	2.3±0.1	2.0±0.1	2.3±0.3	2.9±0.4
20:5ω3	27.7±0.2	27.1±1.2	27.5±0.3	27.4±1.1	24.7±1.2	24.4±1.1	22.7±0.5	22.8±0.5	21.6±1.0	22.6±1.2
22:5ω3	2.2±0.2	1.9±0.1	1.6±0.1	1.8±0.2	2.1±0.2	_	_	2.0±0.4	1.9±0.3	_
22:6ω3	34.7±1.1	36.8±1.2	37.2±1.6	38.4±1.2	38.8±1.1	40.0±4.1	38.1±1.0	39.5±1.3	38.1±3.2	29.2±1.1
other FAs	2.5±0.2	1.9±0.2	2.0±0.2	tr	tr	_	_	tr	tr	_
SFA%	22.0±0.5	19.8±1.3	19.0±0.4	17.5±1.6	20.8±1.3	21.5±2.8	23.5±0.8	23.0±0.8	24.5±3.1	27.1±1.3
MUFA%	10.3±0.3	11.7±1.1	12.0±1.4	13.3±0.5	11.5±1.2	11.8±2.0	13.4±1.1	10.7±0.02	11.6±0.6	18.1±2.5
HUFA%	67.4±0.7	68.1±2.3	68.7±1.3	69.3±2.1	67.7±2.5	66.7±4.9	63.1±0.4	66.3±0.8	63.9±3.7	54.8±1.6

Notably, at intermediate time (6 days) under 15°C and 24°C, the membrane FA composition (PC1score values for membrane lipids) was characterized by large standard deviation values (Fig 2A).

### Storage FA composition

**Fig 3:**
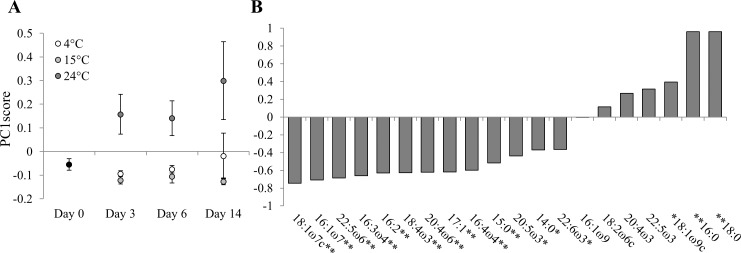
Storage FA composition of the copepod *P*. *littoralis* prior (day 0) and after 3, 6 and 14 days of food deprivation at different temperatures (4, 15 and 24°C). (A) The PC1score values (±SD) for storage lipids are displayed with (B) the Spearman’s rho correlation coefficients of individual FAs (%) used for the PCA with the correlation significance levels (*p <0.05; **p <0.01).

Regarding the original storage FA composition, short-term food deprivation under 15°C and 24°C induced significant modifications (pairwise tests, both p <0.05) (Fig 3A). Moreover, the storage FA composition of copepods exposed to 24°C deviated significantly from 4°C and 15°C (pairwise tests, both p <0.05). Eventually, long-term food deprivation resulted in significant differences in storage FA composition among all temperatures (p <0.01 for temperature; no time or interaction) (pairwise tests, all p <0.05).

At the onset of the experiment, FA associated with storage lipids were rather evenly distributed among PUFA (polyunsaturated FAs i.e. FA with > 1 double bond; 38.5±0.8%), MUFA (36.5±0.8%), HUFA (33.4±0.8%) and SFA (24.9±1.3%) classes (Table 2). Short-term food deprivation under 15°C and 24°C altered the original membrane FA composition, but trends were opposite. Under 15°C, the changes were modest i.e. a subtle increase in HUFA% and decrease in SFA%, caused by an increase in the relative concentrations of 18:1ω7c, 20:4ω6 and 22:5ω6 at the expense of 16:0 (Table 2) (all p <0.01 Fig 3B). Long-term food deprivation under 15°C maintained or magnified these changes (Fig 3A), and additionally, reduced the relative EPA concentration (Table 2) (p <0.05 Fig 3B). Short-term food deprivation under 24°C increased the SFA% (39.9±6.1%) at the expense of the other FA classes (Table 2) and this pattern was caused by the increase in relative concentrations of 16:0, 18:0 at the expense of 16:1ω7 (all p <0.01), EPA and DHA (both p <0.05) (Fig 3B). Prolonged food deprivation under 4°C and 24°C resulted in large standard deviation values of the storage FA composition (PC1score values for storage lipids) (Fig 3A).

**Table 2:** Storage FA composition (% ±SD) of the copepod *P*. *littoralis* prior (day 0) and after 3, 6 and 14 days of food deprivation at different temperatures (4, 15 and 24°C); tr, traces (<1%); other FAs, sum of all FAs <1% in all treatments.

		4°C			15°C			24°C		
(%)	Day 0	Day 3	Day 6	Day 14	Day 3	Day 6	Day 14	Day 3	Day 6	Day 14
14:0	2.5±0.1	2.7±0.2	2.4±0.8	2.6±0.1	2.3±0.2	2.5±0.2	2.6±0.4	2.3±0.4	2.0±0.2	2.2±0.3
16:0	19.2±0.6	18.0±0.2	18.9±0.7	19.6±2.4	17.6±0.6	17.9±0.5	16.7±0.7	25.4±2.4	23.9±2.5	29.6±4.2
16:1ω7	33.0±0.8	34.0±1.3	34.2±0.7	31.9±2.8	33.5±0.7	33.7±1.5	31.7±1.9	26.1±3.7	25.5±2.9	20.7±7.2
16:2	1.5±0.1	1.5±0.1	1.5±0.1	1.3±0.02	1.5±0.03	1.5±0.2	1.3±0.1	1.1±0.1	1.2±0.1	tr
18:0	2.2±0.6	1.8±0.3	2.4±0.4	4.8±3.4	1.3±0.3	2.1±0.9	1.8±0.3	10.7±3.9	10.5±3.6	18.5±9.4
18:1ω9c	tr	tr	tr	tr	1.0±0.1	1.0±0.1	1.1±0.1	1.0±0.1	1.2±0.3	1.4±0.3
18:1ω7c	1.8±0.03	1.9±0.2	1.8±0.2	2.0±0.2	2.4±0.2	2.3±0.1	2.3±0.1	1.6±0.3	1.7±0.3	1.6±0.4
18:4ω3	1.1±0.03	1.1±0.05	1.1±0.1	1.0±0.03	1.2±0.03	1.1±0.1	1.0±0.04	tr	tr	tr
20:4ω6	tr	1.8±0.5	1.2±0.6	1.8±1.3	2.1±0.7	3.1±0.3	6.5±3.6	1.0±0.3	1.4±0.8	1.2±0.3
20:5ω3	27.0±0.6	26.5±1.0	25.6±0.4	24.6±1.4	26.4±0.7	23.5±0.9	21.9±1.3	21.0±1.5	22.1±3.4	15.8±5.0
22:5ω6	tr	1.0±0.03	1.7±0.5	1.7±0.5	1.8±0.4	3.6±1.4	5.0±0.1	tr	tr	tr
22:5ω3	1.2±0.03	1.0±0.04	1.0±0.1	1.0±0.1	1.0±0.05	tr	_	1.0±0.1	1.0±0.2	tr
22:6ω3	3.5±0.1	3.5±0.1	3.3±0.2	3.3±0.2	3.8±0.1	3.3±0.1	3.1±0.3	3.0±0.1	3.2±0.7	2.6±0.7
other FAs	5.2±0.1	4.5±0.1	4.0±0.1	3.5±0.1	4.4±0.7	3.6±0.5	5.1±2.3	4.7±0.1	4.8±0.2	3.7±0.5
SFA%	24.9±1.3	23.1±0.3	24.4±0.7	27.8±5.9	22.0±1.0	23.3±1.0	22.0±1.0	39.9±6.1	37.9±6.6	51.8±13.7
MUFA%	36.5±0.8	37.2±1.4	37.5±0.5	35.2±2.5	37.7±0.8	38.1±1.7	38.1±2.2	29.4±3.8	29.3±2.8	24.5±7.0
PUFA%	38.5±0.8	39.7±1.2	38.1±0.7	37.0±3.5	40.3±0.4	38.6±1.3	39.9±2.4	30.6±2.3	32.8±3.8	23.7±6.9
HUFA%	33.4±0.8	34.5±1.1	33.0±0.5	32.4±3.2	35.3±0.2	34.4±1.2	36.5±2.2	26.8±1.9	29.1±3.4	20.9±5.8

## Discussion

### Role of storage lipids for copepod survival

Starvation resistance depends on the amount of energy reserves and the way a species allocates them to reproduction, growth and metabolism [31,32]. Storage lipids may be composed of wax esters and triacylglycerols (TAG) which serve as long- and short-term energy deposits, respectively. In particular, wax esters are the dominant storage lipid in many deep-living and polar zooplankton taxa [3]. To our knowledge, the presence of wax esters as primary storage lipid has been observed only in one harpacticoid species in association with diapause [16]. Although the storage lipid content comprised a large part of the total FA content in *P*. *littoralis* i.e. 80%, 3 days of food deprivation reduced the storage content substantially, thereby suggesting TAG to be the main storage lipid in this harpacticoid species. Similar short-term storage depletion has been observed in other invertebrates such as *Crassostrea gigas* postlarvae [33] and *Calanus helgolandicus* [34].

When food deprivation occurs, the organism’s response is integrated at all levels of organization and is directed towards survival of the species [32]. The transition from fasting to starvation is thought to occur after massive degradation of adipose tissue to fuel most bodily metabolism [32]. In the current experiment, the transition likely occurred after 3 days of food deprivation as the storage content was substantially reduced under 4°C and 15°C with factors 5 and 13, respectively. However, the drop in storage FA content was not coupled with an increased copepod mortality and consequently, this lag period i.e. from day 3 to day 14 suggested catabolism of other energy sources during starvation. The relative importance of several metabolic reserves and the order of utilization varies among species [1,35]. In some crustaceans, proteins appear the main energy source during food deprivation [36,37] or lipids [38–40] or both simultaneously [36].

In contrast to storage (neutral) lipids, membrane (polar) lipids are usually conserved in food deprived crustaceans due to their role as structural components of cell membranes [1,41,42]. This deviates from the substantial decrease in original membrane FA content, observed after short-term food deprivation. Although these reductions are minor compared with the depletion of storage FA, they diverge from previous observations where the membrane FA content stayed relatively constant in 10-days starved copepods [8]. A higher observation frequency during the first 3 days of food deprivation, capturing the bulk depletion of storage and membrane FA, would have yielded more information on their depletion order and also the depletion rate under different ambient temperatures. The decrease in membrane FA content could have resulted from the degradation of cell organelles as was previously observed in the enterocytes of 4-day starved *Daphnia magna* specimens [31]. In particular, after depletion of whole body resources, the rough endoplasmic reticulum and dictyosome (Golgi complex) were reduced, potentially reflecting the diminished demand for digestive enzymes. Eventually, the cell height was reduced by up to one-fifth of its height. The reduction of membrane FA content was smaller under cold exposure. Higher quantity of intracellular membranes under cold conditions was previously observed and is thought to compensate for the reduced diffusivity constants by reducing the diffusion path length of metabolites [43,44].

Ambient temperature had a clear effect on the net energy balance of starved copepods. In particular, the storage FA content in starved copepods exposed to 4°C remained almost double compared with the 15°C treatments, indicating that the metabolic cost of living increases with temperature [22,45]. However, the lipid response of copepods exposed to heat stress clearly deviated from this concept. At elevated temperature, the organism’s function is limited due to the mismatch between the demand for oxygen by the tissues and the supply of oxygen by the cardiovascular system [46]. Under heat stress, a switch to partial anaerobiosis may occur [47], resulting in a limited decrease of the copepod’s storage FA content. In contrast to carbohydrates and free amino acids that can be oxidized aerobically or anaerobically, lipids are predominantly catabolized in aerobic pathways [48]. This transition to partial anaerobiosis occurs well before the onset of lethal temperatures but heralds a time-limited situation, where temporary survival is restricted to a few days or weeks. The critical temperature that evokes the transition to partial anaerobiosis correlates with the environmental temperatures in the marginal populations of many aquatic ectotherms [48]. The biogeographic range of the harpacticoid *P*. *littoralis* is restricted to Northern Europe [49] and North-America [50] and this identifies *P*. *littoralis* as a temperate species typical for northern latitudes. Given the temperature range in its natural habitat, 4°C to 20–22°C in the Westerschelde estuary [51], the 24°C treatment represented a summer extreme and likely approached the critical temperature, evoking heat stress in the copepod.

### Temperature-dependent storage FA mobilization

Another question that rises is how the FA profile of a consumer changes when lipid stores are mobilized rather than deposited. Currently, no general consensus exists on the issue of differential FA mobilization. *In vitro* studies using white adipocyte cells from rats confirmed selective FA mobilization [52], while no temporal change in overall FA composition was observed in natural long-term fasting studies in several phocid and otariid pinniped pups and juveniles [7]. These divergent responses may indicate different physiological states as some species are confronted with natural cycles of food deprivation, or food deprivation is part of their natural history and they are therefore exposed to fasting rather than starvation [53]. According to [6], FAs are mobilized more readily when they are short, unsaturated and when their double bonds are closer to the methyl end of the chain. Although the exact mechanism of selective FA mobilization remains unclear, hypotheses include selectivity of the rate-limiting enzyme (hormone sensitive lipase) towards storage FA in the process of lipolysis and differential lipid partition of TAG molecules at the lipid-water interphase based on their polarity. From a caloric point of view, longer FA chains allow for more efficient lipid utilization since every FA has to be activated by one ATP for catabolism, independent of its chain length [54].

In the current study, short-term starvation under 15°C and 24°C induced pronounced changes in the original storage FA composition. Despite strong compositional changes under heat stress, lipid depletion was modest. Therefore, the observed compositional changes likely reflected additional heat-stress induced responses. In particular, mitochondrial ROS (reactive oxygen species) formation might increase with temperature, as was demonstrated in a eurythermal marine ectotherm [55]. ROS species have the potential to damage cellular macromolecules such as lipids, proteins and DNA [46]. Among the different FA classes, PUFA are most prone to lipid peroxidation [56,57] and once lipid peroxide radicals are formed, an autocatalytic chain of lipid peroxidation can be initiated. Furthermore, the EPA and DHA in mitochondrial membranes would enhance susceptibility to damage by ROS, generated through oxidative phosphorylation [58]. This process might be linked with the observed drop in relative PUFA, HUFA and in particular EPA, in storage lipids of starved copepods exposed to heat stress. Therefore, evaluation of selective FA mobilization will be restricted to the responses under 4°C and 15°C as these are within the habitat temperature range and were characterized by the most pronounced lipid mobilization. Food deprivation under 15°C evoked the strongest compositional changes in storage FA, in contrast to 4°C, and this indicated the temperature-dependence of selective FA mobilization. Although the proportions of individual FA changed significantly, no clear shifts among FA classes were observed as was the case in other starvation studies focusing on crustaceans. For example, [8] reported the utilization of PUFA and certain MUFA at the expense of SFA in a freshwater calanoid copepod. In contrast, [9] observed preferential PUFA retention and use of MUFA and SFA in an amphipod species during fasting, although this study made no distinction between lipid fractions. Long-term food deprivation maintained or increased the compositional FA changes and therefore identified a time-aspect in the mobilization process. Noteworthy was the lag period of EPA depletion in the storage lipids of copepods. The initial EPA% was maintained during the first days of food deprivation but decreased eventually with smallest reduction under 4°C. The EPA requirement appeared higher under cold conditions which is in line with previous work on *Daphnia pulex* [10]. Long-term food deprivation under the temperature extremes increased the standard error of the copepod storage FA composition. High inter individual variability was previously attributed to the use of individuals from natural populations [8,9] and consequently, might also apply to this study.

### Altered membrane structure and function

The membrane FA composition of *P*. *littoralis* harpacticoids collected from the field was dominated by DHA (±35%), followed by EPA (±30%) and 16:0 (±17%). Although DHA slightly prevailed the membranes compared to EPA, both essential FA were rather equally present. Regarding the membrane FA composition of other first-level consumers, this harpacticoid species can be positioned between cladocerans and calanoid copepods. In particular, DHA (0.9–2.1%) is almost absent in membranes of cladocerans in contrast to EPA (12–23%) [59], while the contribution of DHA (±35%) to the membranes of calanoids (e.g. northern-latitude *Calanus* species [60], the freshwater calanoid *Eudiaptomus gracilis* [8]) is almost twice the contribution of EPA (±17%).

When exposed to fasting, certain organisms might be able to modulate the FA composition of their cell membranes to slow down metabolism and to prolong survival as energy stores progressively decrease [8,61]. In addition, cold stress or heat shock can alter the membrane properties of ectotherms such that, unless they are corrected quickly, damage and possibly, death may occur [62]. Although profound changes in membrane FA composition appeared only after long-term food deprivation, subtle shifts in FA classes were already observed after 3 days. In particular, cold exposure is thought to increase the membrane lipid order but can be compensated by first and second *cis*-double bond insertions in membrane FA [62]. This may explain the increase and decrease of the MUFA% and SFA%, respectively, in the copepod’s membrane under 4°C. Noteworthy was the temperature-dependent EPA% response. Cold exposure conserved EPA, while an immediate decrease was observed under 15°C. Under heat stress, this decrease was even more pronounced. A similar response was observed for storage associated EPA% and this suggests a tight link between both lipid fractions and has been proposed previously [63]. Cell membrane lipids experience a natural turnover [43], thereby acting as an additional driver for storage FA mobilization. Consequently, ambient temperature might indirectly affect the storage FA composition, through the link with membrane FA, although the storage FA composition is mainly affected by the diet [64]. Indeed, mobilized storage FA are also used for the turnover of cell membranes and as precursors of lipid mediators (eicosanoids) [52]. EPA and ARA are precursors of eicosanoids i.e. short-lived hormone-like substances which have opposite effects in inflammation and immune related processes [65,66]. ARA-derived eicosanoids promote inflammation while those from EPA are rather anti-inflammatory [65]. Consequently, it is not surprisingly that the EPA/ ARA ratio decreased profoundly in copepods subjected to food and heat stress. In contrast to the EPA%, short-term food deprivation increased the original DHA% regardless of the temperature. This indicates DHA selective retention and is in line with the findings of [11]. DHA is thought to produce an optimal acyl-chain packing array for the functioning of transmembrane proteins involved in the excitatory response [66]. Moreover, dietary DHA appeared important for the survival, eye development and pigmentation in halibut larvae [13]. Preferential retention of essential FA in unfavorable conditions is of great importance for maintaining the cell’s biochemical competency [9]. However, on the long term, food deprivation combined with heat exposure resulted in a sharp decline of the DHA% and a high copepod mortality. In view of the essential role of DHA for the organism’s health, it is plausible that the sharp decline in the DHA% evoked the increased copepod mortality.

### Ecological significance

The use of energy reserves differs among species and is not only related to the biochemistry and physiology of nutrition but also to the living environment of the crustaceans [1,9]. The harpacticoid *P*. *littoralis* inhabits a temperate, intertidal zone year round and its capacity for thermal acclimatization is therefore expected to be high. Previous research investigating the harpacticoid species assemblage at the Paulina intertidal salt marsh (Westerschelde estuary) suggested that food availability i.e. mainly microphytobenthos, does not limit overall copepod abundance but that shifts in resource composition and/ or other environmental variables drive assemblage composition [21,67]. However, the spatial heterogeneity of microphytobenthos [68–70] in combination with the limited mobility of *P*. *littoralis* and the absence of planktonic larval stages [71] may lead to periodical events of food limitation, especially in view of the selective feeding behavior of harpacticoid copepods [72–74]. Furthermore, occasional food limitation caused by disproportionally high grazing pressure has been reported [75]. *P*. *littoralis* showed some starvation resistance which appeared from the use of storage lipids during the first days of food deprivation and the DHA retention in the copepod’s membrane.

Under heat stress, storage lipids appeared inefficient as energy source and copepods might have switched to partial anaerobiosis which is usually less efficient. Although this response extends the time period of survival, it seriously compromises activities like foraging and reproduction or performances like growth. In the intertidal zone, exposure to heat stress and possibly oxygen deficiency may occur during daytime at low tide for some species [47] but is also time-limited due to the tidal regime. Nevertheless, our results suggest that a rise in oceanic warming, accompanied by increased temperature fluctuations and frequency of temperature extremes [76], might rule out the use of storage lipids as short-term energy source and render *P*. *littoralis* specimen more vulnerable to periods of food limitation.

## References

[1] Sánchez-Paz A, García-Carreño F, Muhlia-Almazán A, Peregrino-Uriarte AB, Hernández-López J, Yepiz-Plascencia G. Usage of energy reserves in crustaceans during starvation: Status and future directions. Insect Biochem Molec. 2006;36: 241–249. 10.1016/j.ibmb.2006.01.002 16551538

[2] Hagen H, Auel H. Seasonal adaptations and the role of lipids in oceanic zooplankton. Zoology. 2001;104: 313–326. 16351846 10.1078/0944-2006-00037

[3] Lee RF, Hagen W, Kattner G. Lipid storage in marine zooplankton. Mar Ecol Prog Ser. 2006;307: 273–306.

[4] Tessier AJ, Henry LL, Goulden CE, Durand MW. Starvation in *Daphnia*: Energy reserves and reproductive allocation. Limnol Oceanogr. 1983;28: 667–676.

[5] Pond DW. The physical properties of lipids and their role in controlling the distribution of zooplankton in the oceans. J Plankton Res. 2012;34: 443–453.

[6] Raclot T. Selective mobilization of fatty acids from adipose tissue triacylglycerols. Prog Lipid Res. 2003;42: 257–288. 12689620 10.1016/s0163-7827(02)00066-8

[7] Iverson SJ. Tracing aquatic food webs using fatty acids In: Arts MT, Brett MT, Kainz MJ, editors. Lipids in Aquatic Ecosystems. Springer Science+Business Media; 2009 pp. 281–307.

[8] Koussoroplis AM, Nussbaumer J, Arts MT, Guschina IA, Kainz MJ. Famine and feast in a common freshwater calanoid: Effects of diet and temperature on fatty acid dynamics of *Eudiaptomus gracilis*. Limnol Oceanogr. 2014;59: 947–958.

[9] Mezek T, Simčič T, Arts MT, Brancelj A. Effect of fasting on hypogean (*Niphargus stygius*) and epigean (*Gammarus fossarum*) amphipods: A laboratory study. Aquat Ecol. 2010;44: 397–408.

[10] Schlechtriem C, Arts MT, Zellmer ID. Effect of temperature on the fatty acid composition and temporal trajectories of fatty acids in fasting *Daphnia pulex* (Crustacea, Cladocera). Lipids. 2006;41: 397–400. 16808154 10.1007/s11745-006-5111-9

[11] Schlechtriem C, Arts MT, Johannsson OE. Effect of Long-term Fasting on the Use of Fatty Acids as Trophic Markers in the Opossum Shrimp *Mysis relicta*—A Laboratory Study. J Great Lakes Res. 2008;34: 143–152.

[12] Virtue P, Nicol S, Nichols PD. Changes in the digestive gland of Euphausia superba during short-term starvation: lipid class, fatty acid and sterol content and composition. Mar Biol. 1993;117: 441–448.

[13] Shields RJ, Bell JG, Luizi FS, Gara B, Bromage NR, Sargent JR. Natural copepods are superior to enriched artemia nauplii as feed for halibut larvae (*Hippoglossus hippoglossus*) in terms of survival, pigmentation and retinal morphology: relation to dietary essential fatty acids. J Nutr. 1999;129: 1186–1194. 10356085 10.1093/jn/129.6.1186

[14] Olsen Y, Evjemo JO, Kjørsvik E, Larssen H, Li K, Overrein I, et al DHA content in dietary phospholipids affects DHA content in phospholipids of cod larvae and larval performance. Aquaculture. 2014;428–429: 203–214.

[15] Bychek EA, Dobson GA, Harwood JL, Guschina IA. *Daphnia magna* can tolerate short-term starvation without major changes in lipid metabolism. Lipids. 2005;40: 599–608. 16149739 10.1007/s11745-005-1421-1

[16] Williams JL, Biesiot PM. Lipids and fatty acids of the benthic marine harpacticoid copepod *Heteropsyllus nunni* Coull during diapause: a contrast to pelagic copepods. Mar Biol. 2004;144: 335–344.

[17] Suderman K, Thistle D. Adult female harpacticoid copepods maintain their energy reserves by feeding while suspended during storms. Mar Ecol Prog Ser. 1998;164: 245–252.

[18] Hicks GRF, Coull BC. The ecology of marine meiobenthic harpacticoid copepods. Oceanogr Mar Biol Annu Rev. 1983;21: 67–175.

[19] Arts MT, Ackman RG, Holub BJ. "Essential fatty acids" in aquatic ecosystems: a crucial link between diet and human health and evolution. Can J Fish Aquat Sci. 2001;58: 122–137.

[20] De Troch M, Boeckx P, Cnudde C, Van Gansbeke D, Vanreusel A, Vincx M et al Bioconversion of fatty acids at the basis of marine food webs: insights from a compound-specific stable isotope analysis. Mar Ecol Prog Ser. 2012;465: 53–67.

[21] Cnudde C. Trophic ecology of intertidal harpacticoid copepods, with emphasis on their interactions with bacteria Ghent University (UGent); 2013.

[22] Gillooly JF, Brown JH, West GB, Savage VM, Charnov EL. Effects of size and temperature on metabolic rate. Science. 2001;293: 2248–2251. 11567137 10.1126/science.1061967

[23] Dalsgaard J, St John M, Kattner G, Müller-Navarra D, Hagen W. Fatty acid trophic markers in the pelagic marine environment. Adv Mar Biol. 2003;46: 225–340. 14601414 10.1016/s0065-2881(03)46005-7

[24] Findlay RH, King GM, Watling L. Efficacy of phospholipid analysis in determining microbial biomass in sediments. Appl Environ Microbiol. 1989;55: 2888–2893. 16348051 10.1128/aem.55.11.2888-2893.1989 PMC203186

[25] Christie WW. Gas Chromatography and Lipids: a practical guide 1st ed. The Oily Press Ltd; 1989.

[26] Boschker HTS, de Brouwer JFC, Cappenberg TE. The contribution of macrophyte-derived organic matter to microbial biomass in salt-marsh sediments: Stable carbon isotope analysis of microbial biomarkers. Limnol Oceanogr. 1999;44: 309–319.

[27] Abdulkadir S, Tsuchiya M. One-step method for quantitative and qualitative analysis of fatty acids in marine animal samples. J Exp Mar Biol Ecol. 2008;354: 1–8.

[28] Eder K. Gas Chromatographic analysis of fatty acid methyl esters. J Chromatogr B. 1995;671: 113–131. 10.1016/0378-4347(95)00142-6 8520689

[29] Clarke K, Gorley RN. Primer v6: User Manual/Tutorial. Plymouth, PRIMER-E 2006.

[30] Van Dooremalen C, Ellers J. A moderate change in temperature induces changes in fatty acid composition of storage and membrane lipids in a soil arthropod. J Insect Physiol. 2010;56: 178–187. 10.1016/j.jinsphys.2009.10.002 19835878

[31] Elendt B, Storch V. Starvation-induced alterations of the ultrastructure of the midgut of *Daphnia magna* Straus, 1820 (Cladocera). J Crustacean Biol. 1990;10: 79–86.

[32] Wang T, Hung CCY, Randall DJ. The comparative physiology of food deprivation: from feast to famine. Annu Rev Physiol. 2006;68: 223–251. 16460272 10.1146/annurev.physiol.68.040104.105739

[33] García-Esquivel Z, Bricelj MV, Felbeck H. Metabolic depression and whole-body response to enforced starvation by *Crassostrea gigas* postlarvae. Comp Biochem Physiol Part A. 2002;133: 63–77. 10.1016/s1095-6433(02)00112-5 12160873

[34] Lee RF, Nevenzel JC, Paffenhöffer GA, Benson AA. The metabolism of wax esters and other lipids by the marine copepod, *Calanus helgolandicus*. J Lipid Res. 1970;11: 237–240. 5441249

[35] Hervant F, Mathieu J, Barré H. Comparative study on the metabolic responses of subterranean and surface-dwelling amphipods to long-term starvation and subsequent refeeding. J Exp Biol. 1990;202: 3587–3895. 10.1242/jeb.202.24.3587 10574735

[36] Mayzaud P. Respiration and nitrogen excretion of zooplankton. IV. The influence of starvation on the metabolism and the biochemical composition of some species. Mar Biol. 1976;37: 47–58.

[37] Johnston DJ, Ritar AJ, Thomas CW. Digestive enzyme profiles reveal digestive capacity and potential energy sources in fed and starved spiny lobster (*Jasus edwardsii*) phyllosoma larvae. Comp Biochem Physiol Part B. 2004;138: 137–144. 10.1016/j.cbpc.2004.02.013 15193268

[38] Evjemo JO, Danielsen TL, Olsen Y. Losses of lipid, protein and n−3 fatty acids in enriched *Artemia franciscana* starved at different temperatures. Aquaculture. 2001;193: 65–80.

[39] Båmstedt U, Holt M. Experimental studies on the deep-water pelagic community of Korsfjorden, Western Norway. Prey-size preference and feeding of *Euchaeta norvegica* (Gopepoda). Sarsia. 1978;63: 225–236.

[40] Ritar AJ, Dunstan GA, Crear BJ, Brown MR. Biochemical composition during growth and starvation of early larval stages of cultured spiny lobster (*Jasus edwardsii*) phyllosoma. Comp Biochem Physiol Part A. 2003;136: 353–370. 10.1016/s1095-6433(03)00167-3 14511754

[41] Arts MT. Lipids in Freshwater Zooplankton: Selected Ecological and Physiological Aspects In: Arts MT, Wainmann BC, editors. Lipids in Freshwater Ecosystems. Springer Science+Business Media; 1999 pp. 71–90.

[42] Helland S, Nejstgaard JC, Fyhn HJ, Egge JK, Båmstedt U. Effects of starvation, season, and diet on the free amino acid and protein content of *Calanus finmarchicus* females. Mar Biol. 2003;143: 297–306.

[43] Hazel JR, Williams EE. The role of alterations in membrane lipid composition in enabling physiological adaptation of organisms to their physical environment. Prog Lipid Res. 1990;29: 167–227. 2131463 10.1016/0163-7827(90)90002-3

[44] Tyler S, Sidell B. Changes in mitochondrial distribution and diffusion distances in muscle of goldfish upon acclimation to warm and cold temperatures. J Exp Zool. 1984;232: 1–9.

[45] Brown JH, Gillooly JF, Allen AP, Savage VM, West GB. Toward a metabolic theory of ecology. Ecology. 2004;85: 1771–1789.

[46] Schulte PM. The effects of temperature on aerobic metabolism: towards a mechanistic understanding of the responses of ectotherms to a changing environment. J Exp Biol. 2015;218: 1856–1866. 10.1242/jeb.118851 26085663

[47] Pörtner HO. Integrating climate related stressor effects on marine organisms: unifying principles linking molecule to ecosystem-level changes. Mar Ecol Prog Ser. 2012;470: 273–290.

[48] Sokolova IM, Frederich M, Bagwe R, Lannig G, Sukhotin AA. Energy homeostasis as an integrative tool for assessing limits of environmental stress tolerance in aquatic invertebrates. Mar Environ Res. 2012;79: 1–15. 10.1016/j.marenvres.2012.04.003 22622075

[49] Veit-Köhler G, De Troch M, Grego M, Bezerra TN, Bonne W, De Smet G et al Large-scale diversity and biogeography of benthic copepods in European waters. Mar Biol. 2010;157: 1819–1835.

[50] Lang K. Monographie der Harpacticoiden Vol. 2 Haran Ohlsson Stockholm; 1948.

[51] Sahan E, Sabbe K, Creach V, Hernandez-Raquet G, Vyverman W, Stal LJ et al Community structure and seasonal dynamics of diatom biofilms and associated grazers in intertidal mudflats. Aquat Microb Ecol. 2007;47: 253–266.

[52] Raclot T, Groscolas R. Selective mobilization of adipose tissue fatty acids during energy depletion in the rat. J Lipid Res. 1995;36: 2164–2173. 8576642

[53] Castellini MA, Rea LD. The biochemistry of natural fasting at its limits. Cell Mol Life Sci. 1992;48: 575–582. 10.1007/BF01920242 1612138

[54] Kattner G, Hagen W. Lipids in marine copepods: Latitudinal characteristics and perspectives to global warming In: Arts MT, Brett MT, Kainz MJ, editors. Lipids in Aquatic Ecosystems. Springer Science+Business Media; 2009 pp. 257–280.

[55] Abele D, Heise K, Pörtner HO, Puntarulo S. Temperature-dependence of mitochondrial function and production of reactive oxygen species in the intertidal mud clam *Mya arenaria*. J Exp Biol. 2002;205: 1831–1841. 12077159 10.1242/jeb.205.13.1831

[56] Hulbert AJ, Kelly MA, Abbott SK. Polyunsaturated fats, membrane lipids and animal longevity. J Comp Physiol B. 2014;184: 149–166. 10.1007/s00360-013-0786-8 24129945

[57] Pamplona R, Barja G, Portero-Otín M. Membrane fatty acid unsaturation, protection against oxidative stress and maximum life span. A homeoviscous-longevity adaptation? Ann NY Acad Sci. 2002;959: 475–490. 11976221 10.1111/j.1749-6632.2002.tb02118.x

[58] Chapkin RS, Hong MY, Fan YY, Davidson LA, Sanders LM, Henderson CE et al Dietary n-3 PUFA alter colonocyte mitochondrial membrane composition and function. Lipids. 2002;37: 193–199. 11908911 10.1007/s11745-002-0880-8

[59] Persson J, Vrede T. Polyunsaturated fatty acids in zooplankton: variation due to taxonomy and trophic position. Fresh Biol. 2006;51: 887–900.

[60] Kattner G, Hagen W. Lipids in marine copepods: latitudinal characteristics and perspective to global warming In: Arts MT, Brett MT, Kainz MJ, editors. Lipids in Aquatic Ecosystems. Springer Science+Business Media; 2009 pp. 257–280.

[61] Stuart JA, Gillis TE, Ballantyne JS. Compositional correlates of metabolic depression in the mitochondrial membranes of estivating snails. Am J Physiol Regul Integr Comp Physiol. 1998;275: 1977–1982. 10.1152/ajpregu.1998.275.6.R1977 9843887

[62] Guschina IA, Harwood JL. Mechanisms of temperature adaptation in poikilotherms. FEBS Letters. 2006;580: 5477–5483. 16824520 10.1016/j.febslet.2006.06.066

[63] Lee RF, Barnes AT. Lipids in the mesopelagic copepod, *Gaussia princeps*. Wax ester utilization during starvation. Comp Biochem Physiol. 1975;52: 265–268. 10.1016/0305-0491(75)90063-2 1175354

[64] Brett MT, Müller-Navarra DC, Persson J. Crustacean zooplankton fatty acid composition In: Arts MT, Brett MT, Kainz MJ, editors. Lipids in Aquatic Ecosystems. Springer Science+Business Media; 2009 pp. 115–146.

[65] Arts MT, Kohler CC. Health and condition in fish: the influence of lipids on membrane competency and immune response In: Arts MT, Brett MT, Kainz MJ, editors. Lipids in Aquatic Ecosystems. Springer Science+Business Media; 2009 pp. 237–255.

[66] Parrish CC. Essential fatty acids in aquatic food webs In: Arts MT, Brett MT, Kainz MJ, editors. Lipids in Aquatic Ecosystems. Springer Science+Business Media; 2009 pp. 309–326.

[67] Cnudde C, Moens T, Werbrouck E, Lepoint G, Van Gansbeke D, De Troch M. Trophodynamics of estuarine intertidal harpacticoid copepods based on stable isotope composition and fatty acid profiles. Mar Ecol Prog Ser. 2015;524: 225–239.

[68] Blanchard GF. Overlapping microscale dispersion patterns of meiofauna and microphytobenthos. Mar Ecol Prog Ser. 1990;68: 101–111.

[69] Jesus B, Brotas V, Marani M, Paterson DM. Spatial dynamics of microphytobenthos determined by PAM fluorescence. Estuar Coast Shelf S. 2005;65: 30–42.

[70] Combe JP, Launeau P, Carrère V, Despan D, Méléder V, Barillé L et al Mapping microphytobenthos biomass by non-linear inversion of visible-infrared hyperspectral images. Remote Sens Environ. 2005;98: 371–387.

[71] Giere O. Meiobenthology, the Microscopic Fauna in Aquatic Sediments. Springer-Verlag Berlin; 1993.

[72] Vanden Berghe W, Bergmans M. Differential food preferences in three co-occurring species of Tisbe (Copepoda, Harpacticoida). Mar Ecol Prog Ser. 1981;4: 213–219.

[73] Nilsson P. Demography of *Mesochra lilljeborgi* and *Amonardia normani* (Copepoda: Harpacticoida) reared on two diatom diets. Mar Ecol Prog Ser. 1987;39: 267–274.

[74] Wyckmans M, Chepurnov VA, Vanreusel A, De Troch M. Effects of food diversity on diatom selection by harpacticoid copepods. J Exp Mar Biol Ecol. 2007;345: 119–128.

[75] Azovsky AI, Saburova MA, Chertoprood ES, Polikarpov IG. Selective feeding of littoral harpacticoids on diatom algae: Hungry gourmands? Mar Biol. 2005;148: 327–337.

[76] Sokolova IM, Lannig G. Interactive effects of metal pollution and temperature on metabolism in aquatic ectotherms: Implications of global climate change. Clim Res. 2008;37: 181–201.

